# Multiple Neural Networks Malfunction in Primary Blepharospasm: An Independent Components Analysis

**DOI:** 10.3389/fnhum.2017.00235

**Published:** 2017-05-10

**Authors:** Xiao-Feng Huang, Meng-Ru Zhu, Ping Shan, Chen-Hui Pei, Zhan-Hua Liang, Hui-Ling Zhou, Ming-Fei Ni, Yan-Wei Miao, Guo-Qing Xu, Bing-Wei Zhang, Ya-Yin Luo

**Affiliations:** ^1^Department of Neurology and Psychiatry, First Affiliated Hospital of Dalian Medical UniversityDalian, China; ^2^Department of Seven Year System, China Medical UniversityShenyang, China; ^3^VIP Ward, First Affiliated Hospital of Dalian Medical UniversityDalian, China; ^4^Department of Image, First Affiliated Hospital of Dalian Medical UniversityDalian, China; ^5^Department of Psychology, Dalian Medical UniversityDalian, China

**Keywords:** blepharospasm, focal dystonia, resting-state fMRI, independent component analysis, sensorimotor integration, right fronto-parietal network, salience network

## Abstract

Primary blepharospasm (BPS) is a focal dystonia characterized by involuntary blinking and eyelid spasms. The pathophysiology of BPS remains unclear. Several neuroimaging studies have suggested dysfunction of sensory processing and sensorimotor integration, but the results have been inconsistent. This study aimed to determine whether patients with BPS exhibit altered functional brain connectivity and to explore possible correlations between these networks and clinical variables. Twenty-five patients with BPS and 25 healthy controls were enrolled. We found that the patient group exhibited decreased connectivity within the sensory-motor network (SMN), which involved regions of the bilateral primary sensorimotor cortex, supplementary motor area (SMA), right premotor cortex, bilateral precuneus and left superior parietal cortex. Within the right fronto-parietal network, decreased connections were observed in the middle frontal gyrus, dorsal lateral prefrontal cortex and inferior frontal gyrus. Regarding the salience network (SN), increased connectivity was observed in the left superior frontal gyrus and middle frontal gyrus. These findings suggest the involvement of multiple neural networks in primary BPS.

## Introduction

Primary blepharospasm (BPS) is a type of focal dystonia that is characterized by persistent or intermittent excessive involuntary blinking and eyelid spasms and has a disabling effect on work and everyday activities and may cause social embarrassment and catastrophic traffic accidents. While the symptomatology of BPS is well defined, its pathophysiology remains unknown. Current theories about the pathophysiology of dystonia are largely based on studies of focal hand dystonia (FHD). Despite some clinical overlap and electrophysiological similarities, the pathophysiology of BPS is likely to be different (Battistella et al., 2017).

Although BPS is classified as a movement disorder, various non-motor symptoms have been reported, including sensory deficits (such as dry eyes, photophobia and eye pain), emotional deficits (such as depression and anxiety) and cognitive deficits (Hall et al., 2006; Alemán et al., 2009; Emoto et al., 2010; Fontenelle et al., 2011; Peckham et al., 2011; Hwang, 2012; Huang et al., 2015). In a previous study, we found that 73% of the patients had emotional incentives (such as high pressure at work, life stress and death of relatives) before onset (Huang et al., 2015), and 86% of them experienced “sensory tricks”. Sensory tricks are a characteristic sensory phenomenon of BPS, and this term refers to the use of tactile stimuli to relax the involved muscles. The mechanism of sensory tricks is speculated to modulate abnormal sensory-motor processing. Recent magnetic resonance imaging (MRI) and electroneurophysiology studies have mapped selected components of neural networks in patients with BPS, with the cumulative evidence suggesting that BPS may represent a network disorder (Dresel et al., 2011; Suzuki et al., 2011; Battistella et al., 2017). Task-related network changes in BPS were related to the sensorimotor network (SMN), including the primary and secondary somatosensory regions (Dresel et al., 2011). A structural neuroimaging study has reported the involvement of the bilateral sensorimotor cortex and anterior cingulated cortex (Suzuki et al., 2011). Another study examining resting state networks in patients with BPS suggested decreased functional connection within the sensorimotor and frontoparietal networks (Battistella et al., 2017).

In recent years, functional magnetic resonance imaging (fMRI) has been accepted as an effective tool to investigate changes in brain function in BPS. Resting-state networks, which are based on measuring intrinsic low frequency physiological fluctuations of the blood oxygen level-dependent (BOLD) signal, reflect the organization of both structural and task-related functional brain networks (Biswal et al., 1995; Damoiseaux and Greicius, 2009; Smith et al., 2009). In contrast to task-related fMRI, for resting-state fMRI, BOLD signals are collected during resting wakefulness without any task-related confounder. Because the explanation of functional changes in BPS may sometimes be ambiguous due to the combination of motor and sensory components, examination of the resting-state functional networks is believed to provide a more uniform and coherent understanding of network alterations. Several resting-state fMRI studies have been conducted on patients with BPS, but the results have differed (Schmidt et al., 2003; Yang et al., 2013; Zhou et al., 2013). Furthermore, these studies only focused on focal brain regions and thus could not reveal abnormal connectivity within whole functional networks of the brain. To clarify changes in functional connectivity, one can apply network analysis based on independent component analysis (ICA) on BOLD time series obtained with resting state fMRI. ICA extracts spatiotemporal patterns of underlying signal components, assuming the components are statistically independent (Beckmann et al., 2005). It has been shown that several important resting state networks, such as the SMN, default mode network (DMN) and executive control network (ECN), can be obtained with high reliability across individuals and groups (Beckmann et al., 2005; Damoiseaux et al., 2006; Smith et al., 2009). In this study, we used ICA to investigate the alterations in functional connectivity in patients with BPS. Based on previous studies (Dresel et al., 2011; Huang et al., 2015; Battistella et al., 2017), we hypothesized that functional brain networks in BPS undergo widespread re-organization.

## Subjects and Methods

### Patients and Controls

A total of 50 participants were recruited for this study, including 25 patients with BPS and 25 age- and gender-matched healthy controls (HCs), from the Neurology Department of the First Affiliated Hospital of Dalian Medical University. All subjects were right-handed according to the Edinburgh Inventory. The diagnoses of BPS were established based on published criteria by a neurologist with long-term experience in movement disorders (Hallett et al., 2008). Known causes of secondary dystonia were excluded based on medical and drug histories, neurological examination, laboratory investigation and conventional MRI. All patients were free of other neurological abnormalities and family history of movement disorders. The severity of BPS in all patients was assessed according to the Jankovic Rating Scale (JRS) immediately before MRI. Disease durations were calculated from the time of symptom onset to the scan date in months. None of the patients used any medications for 24 h prior to MRI. This study was carried out in accordance with the recommendations of Declaration of Helsinki, First Affiliated Hospital of Dalian Medical University with written informed consent from all subjects. All subjects gave written informed consent in accordance with the Declaration of Helsinki. The protocol was approved by the First Affiliated Hospital of Dalian Medical University. None of the patients had received botulinum neurotoxin (BoNT) treatment within 3 months prior to the first MRI scan. Four of the patients got a second MRI scan at about 50 days after BoNT treatment, when the spasm was suppressed (total JRS score ≤ 1).

### MRI Acquisition Protocol

All images were acquired with a GE Signa HDxt America 3.0 T scanner using a 32-channel head coil. Earplugs were used, and movement was minimized by stabilizing the head with cushions. High-resolution T1-weighted images were acquired via a volumetric three-dimensional spoiled gradient recall sequence (TR = 3.7 ms, TE = 1 ms, slice thickness = 6.0 mm). Functional images (gradient-echo EPI, TR = 3000 ms, TE = 30 ms, flip angle = 90°, FOV: 64 × 64 mm, 32 axial slices, slice thickness = 4 mm, gap = 0 mm, 105 scans, 5 dummy scans, total acquisition time: 5 min 15 s) were acquired with the participants’ eyes closed. The participants were instructed to “relax with eyes closed and not think about anything in particular”. Adherence to this instruction was confirmed in a post-scanning debriefing.

### MRI Analysis

All fMRI data preprocessing and statistical analyses were performed with the Data Processing Assistant for Resting-State fMRI (DPARSF; Chao-Gan and Yu-Feng, 2010)^1^ which is based on Statistical Parametric Mapping (SPM8)^2^ on the Matlab platform. The first five volumes of the functional images were removed for signal equilibration and the adaptation of the participants to the scanning environment. The remaining EPI images were preprocessed using the following steps: slice timing, motion correction, spatial normalization to the standard Montreal Neurological Institute (MNI) EPI template in SPM8 with resampling to 3 × 3 × 3 mm^3^, and spatial smoothing with a 6-mm full-width at half-maximum (FWHM) Gaussian kernel. Based on the head motion records within each fMRI run, no participant exhibited greater than 1.5 mm of maximum displacement in the *x*, *y* or *z* direction or greater than 1° of angular rotation about any axis.

Group ICA was performed with the GIFT toolbox (GIFT v2.0^3^) using the Infomax algorithm (Bell and Sejnowski, 1995) and standard PCA and back-reconstruction using the GICA method. For each subject, 36 independent components were extracted. All single-subject component maps from all subjects were then “clustered” at the group level, which resulted in 36 single-group average maps that were visually inspected to determine the main physiological resting-state networks. The selection of clusters of interest implied the presence of anatomically relevant areas in each group component map that reproduced the layouts of the main physiological RSN jointly and consistently across subjects. Network co-activation differences between patients with BPS and HCs were examined using REST (v1.8; Song et al., 2011) with two-sample *t*-tests performed on the spatial distributions of the components. Statistical images were AlphaSim corrected (*p* < 0.05).

## Results

### Clinical Data

The clinical and demographic characteristics of the samples and levels of significance of the clinical variables are provided in Table 1. There were no significant differences in the demographic variables between the patients with BPS and the HCs.

**Table 1:** **Demographic and clinical characteristics of patient group and control group**.

	P	C	*P*-Value
Age (years)	56.28 ± 1.89	55.17 ± 1.69	0.67
Gender (M:F)	25 (8:17)	25 (8:17)	>0.99
Education (years)	9.70 ± 1.37	8.00 ± 1.39	0.41
Disease duration (months)	56.36 ± 10.67	None	–
JRS	6.36 ± 0.33	None	–
HAMA	9.42 ± 1.86	2.00 ± 0.37	<0.001
Familly history	None	None	–

*C, controls; P, patients; M, male; F, female; JRS, Janckovic Rating Scale; HAMA, Hamilton Anxiety Scale*.

### Sensory-Motor Network

**Figure 1:**
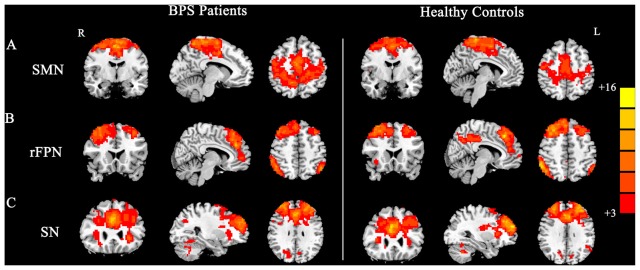
**Group maps of the networks showing statistically significant differences between patients and controls: (A)** sensorimotor network (SMN), **(B)** right frontoparietal network (rFPN), **(C)** salience network (SN).

**Figure 2:**
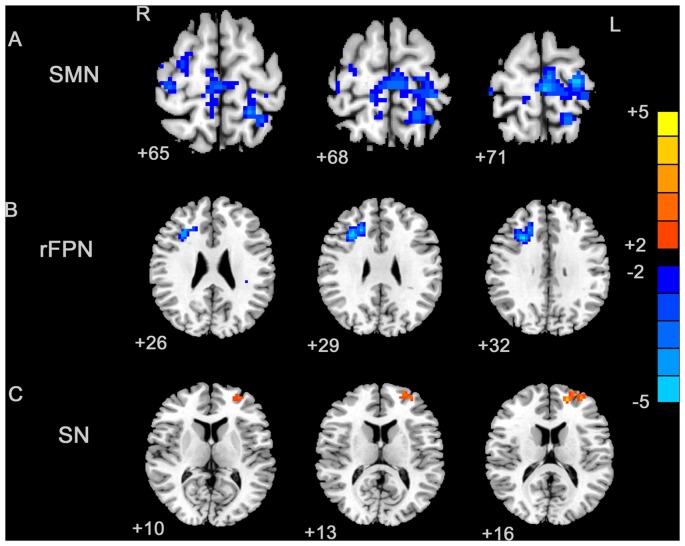
**Between-group effects in the SMN, rFPN and primary visual network (PVN)**. The between-group effects for three networks are shown. The between-group effects were AlphaSim corrected (*p* < 0.05). **(A)** Precentral regions, postcentral regions, frontal regions, supplementary motor area (SMA), precuneus and parietal regions that were abnormally connected within the SMN, indicating decreased connectivity within the blepharospasm (BPS) group. **(B)** The brain regions linked to the rFPN and exhibiting decreased connectivity in the BPS group. **(C)** The SN exhibited a BPS-related increase in the connectivity of several regions, including the left superior frontal area, middle frontal area (including dorsolateral prefrontal cortex (DLPFC)).

By visually inspecting the ICA-derived components of the RS-fMRI data from the two groups, we identified several RSN components using similar methodology to previous studies (Delnooz et al., 2013; Battistella et al., 2017). Between-group ICA revealed significant distinct functional connectivity abnormalities of the SMN and right frontoparietal network (rFPN) in the patients compared with those in the HCs (Figure 1). Generally, the SMN includes the sensorimotor cortex, supplementary motor area (SMA) and secondary somatosensory cortex and closely corresponds to the brain activation that occurs during action execution and perception (Beckmann et al., 2005; Smith et al., 2009). Compared with those in healthy participants, patients with BPS showed decreased functional connectivities in the bilateral primary sensorimotor cortex, SMA, right superior frontal gyrus (premotor cortex), bilateral precuneus and left superior parietal cortex (Alphasim corrected *P* < 0.05, cluster size >85 voxels, cluster edge connected; Figure 2A; Table 2).

**Table 2:** **Local maxima of regions with altered connectivity within the sensorimotor network (SMN)**.

Network	Contrast	Region	Area	*X*	*Y*	*Z*	*T*-Score
Sensorimotor network	P < C	Precentral cortex_R	4	36	−27	66	−3.22
		Premotor cortex_R	6	33	54	16	−3.16
		SMA_R	6	7	−15	63	−2.47
		Precuneus_R	5	6	−47	68	−2.52
		Superior parietal_L	2/5/7	−22	−45	69	−3.70
		Precentral cortex_L	4/6	−27	−21	72	−4.28
		Precuneus_L	5	−15	−47	68	−2.53
		Postcentral cortex_L	2/3	−18	−44	69	−3.29
		Paracentral Lobule_L	4/6	−2	−25	72	−3.80

*C, controls; P, patients; R, right; L, left; SMA, supplementary motor area. Between-group effects are corrected for Alphasim (*P* < 0.05, cluster size >85 voxels, cluster edge connected)*.

### Right Frontoparietal Network

The rFPN has been found to play important roles in cognitive, emotional and pain information processing (Smith et al., 2009). The rFPN showed significant group differences in the middle frontal gyrus, dorsolateral prefrontal cortex (DLPFC) and inferior frontal gyrus (Alphasim corrected *P* < 0.05, cluster size >85 voxels, cluster edge connected; Figure 2B; Table 3). No significant differences were found for the CN. The control vs. patient analysis at *t* = 2 confirmed the patterns of altered connectivity (data not shown).

**Table 3:** **Local maxima of regions with altered connectivity within the right frontoparietal network (rFPN)**.

Network	Contrast	Region	Area	*X*	*Y*	*Z*	*T*-Score
Right fronto-parietal network	P < C	Middle frontal gyrus_R	46/48	30	27	31	−4.13
		DLPFC_R	9	20	27	34	−3.28
		Inferior frontal gyrus_R	48/44	30	27	29	−3.62

*C, controls; P, patients; R, right; DLPFC, dorsolateral prefrontal cortex. Between-group effects are corrected for Alphasim (*p* < 0.05, cluster size >85 voxels, cluster edge connected)*.

### Salience Network

We explored four other RSNs that were derived from Smith et al. (2009), i.e., the SN, the left frontoparietal network (LFPN), the auditory network (AN) and the primary visual network (PVN) and applied an AlphaSim corrected *p* < 0.05. Only the SN exhibited differential connectivity. Between-group analysis showed increased connectivity in patients with BPS in the left superior frontal gyrus and middle frontal gyrus (including the DLPFC; Table 4; Figure 2C).

**Table 4:** **Local maxima of regions with altered connectivity within the salience network (SN)**.

Network	Contrast	Region	Area	*X*	*Y*	*Z*	*T*-Score
Salience network	P > C	Superior frontal gyrus_L	10	−18	54	18	4.128
		Middle frontal gyrus_L/ DLPFC	10/46	−33	54	16	3.04

*C, controls; P, patients; L, left; DLPFC, dorsolateral prefrontal cortex. Between-group effects are corrected for Alphasim (*p* < 0.05, cluster size >85 voxels, cluster edge connected)*.

### Correlation Analysis

**Figure 3:**
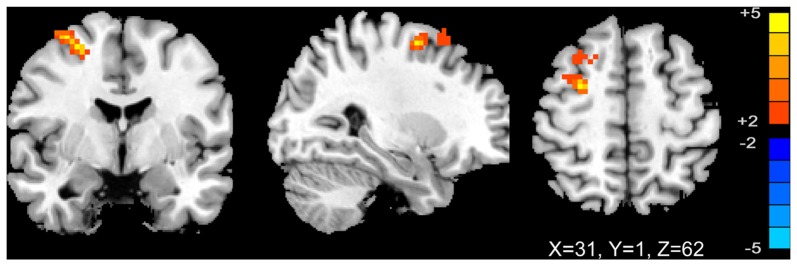
**T-map of group-level sensory-motor network connectivity in ST(+) and ST(−) patients (*p* < 0.01, AlphaSim corrected)**. ST(+) patients demonstrated higher connectivity in right premotor cortex (superior frontal gyrus and middle frontal gyrus BA 6).

**Figure 4:**
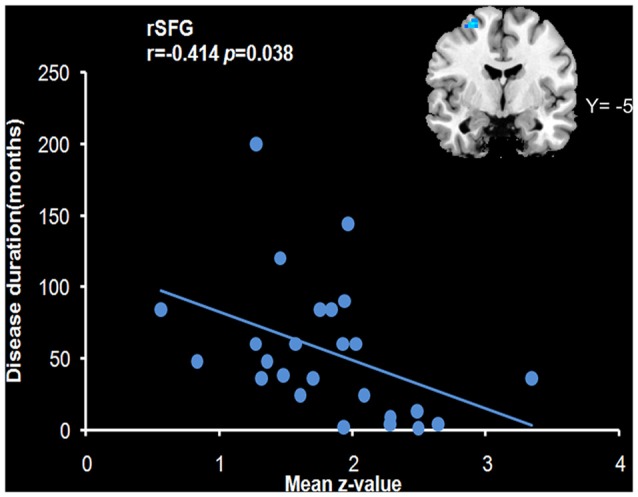
**Correlation with the mean *z*-value of the right superior frontal gyrus (rSFG)**. The results revealed a negative correlation of the mean *z*-value of the rSFG with disease duration (*r* = −0.414, *p* = 0.038).

We analyzed the correlations of the abnormal regions within the SMN (Table 2) with disease characteristics. Among the 25 subjects, 12 were sensory tricks-positive (ST+), 9 did not perform sensory tricks (ST−) and the other 4 were uncertain. ST+ patients exhibited significant higher connectivity in the right premotor cortex compared to ST− patients (superior frontal gyrus and middle frontal gyrus BA 6; Figure 3; Table 5). The results also showed a significant negative correlation between the rSFG and disease duration (Pearson’s correlation *r* = −0.414, *p* = 0.038; Figure 4). In addition, we observed a positive correlation between the left superior frontal gyrus and HAMA scores, but this result was not significant (Pearson’s correlation *r* = 0.508, *p* = 0.092).

**Table 5:** **Different regions within the SMN between ST(+) and ST(−) patients**.

Network	Contrast	Region	Area	*X*	*Y*	*Z*	*T*-Score
Sensori-motor network	ST(+) > ST(−)	Superior frontal gyrus_R	6	27	−3	57	4.998
		Middle frontal gyrus_R	-	-	-	-	-

*ST, sensory tricks; R, right. Between-group effects are corrected for Alphasim (*p* < 0.01, cluster size >19 voxels, cluster edge connected)*.

### Treatment-Related Connectivity

We compared the connectivity maps from before and after treatment, evaluating BoNT-driven connectivity changes. There were changes in the SMN and rFPN, but neither persisted through AlphaSim correction. Regarding the SMN, increased connectivity was found after treatment in the left SMA and right premotor cortex, and decreased connectivity was found in the right SMA and right precentral gyrus. The right inferior parietal cortex (BA 48), middle frontal gyrus (BA 46), superior frontal gurus (BA 8) and middle temporal gurus (BA 20) demonstrated increased connectivity within rFPN after BoNT injections.

## Discussion

In this study, the patient group showed decreased functional connectivity in the SMA and premotor cortex within the SMN. The functions of the SMN are primarily related to sensory processing, motor planning and motor execution. Specifically, the SMA and premotor cortex seems to play critical roles in motor preparation and execution during the construction of a motor representation and is important in the control of the engagement in motor inhibition and halting or overriding of motor responses(Carbonnell et al., 2004; Gross et al., 2005; Tanji and Hoshi, 2008). Decreased connectivity of the SMA has been previously been linked to abnormal inhibition in patients with focal dystonia (Naumann et al., 2000; Jin et al., 2011). The premotor cortex showed abnormal connectivity with the primary motor cortex, parietal cortex and basal ganglia and improved deficits in reciprocal inhibition and mitigation of spasms following stimulation of this region (Kranz et al., 2009; Pirio Richardson, 2015). In this study, patients that were sensory tricks-positive exhibited higher connectivity in the premotor cortex, which suggested a relative reserved function for this area and a central role for the premotor cortex in the mechanism of sensory tricks. Our finding of a significant relationship between the decreased connectivity in the rSFG and the duration of disease suggests that impairment of this region may be a secondary manifestation of dystonic symptoms, whereas deficiencies in other regions (e.g., the SMA and sensory cortex) may represent primary deficiencies.

Decreased connectivity in the sensory cortex suggests deficits in sensory processing play a role in abnormal sensorimotor integration. Previous studies showing electrophysiology and structural changes in the primary somatosensory cortex support the concept of abnormal sensory-motor integration in BPS (Martino et al., 2011; Suzuki et al., 2011; Yang et al., 2013). The findings of our study extend current knowledge by providing functional neuroimaging evidence for the presence of sensory alterations at the network level. In the present study, the superior parietal cortex within the SMN also exhibited decreased functional connectivity. The parietal cortex, particularly the posterior parietal cortex, serves as an important sensory-associative area that integrates somatosensory, visual and spatial information to create a body scheme prior to the execution of voluntary movements (Sereno and Huang, 2014). Decreases in gray and white matter integrity in the parietal lobes of patients with BPS have been observed via voxel-based morphometry (VBM) and diffusion tensor imaging (DTI) analyses (Etgen et al., 2006; Yang et al., 2014), respectively. Additionally, infarction lesions in the parietal cortex can induce BPS (Jacob and Chand, 1995). These findings may be representative of impairment in the integration of sensory information with movement processing. These results suggest that faulty processing of motor programs in patients with BPS is possibly related to a larger planning defect that results in difficulty focusing a motor command on the appropriate muscles.

In this study, the rFPN showed significant group differences in the middle frontal gyrus, DLPFC and inferior frontal gyrus. The fronto-parietal (or “executive-attention”) network seems to be critical for cognitive control and complex attention control, and it includes regions such as the dorsal frontal and parietal cortices, which are known to mediate cognitive and executive control processing. Moreover, rFPN dysfunction may be involved in abnormal processing of harmful external stimuli (Tan et al., 2015). Numerous studies have demonstrated that patients with BPS exhibit relatively poor performance on non-motor tasks related to cognition functional domains, for example, complex movement planning, visuo-spatial working memory, tactile recognition and sustained attention (Scott et al., 2003; Alemán et al., 2009). If BPS disrupts normal pain processing by the rFPN, this dysfunction may be a strong contributor to central nervous system-mediated sensory dysfunction. Delnooz et al. (2013) explored rFPN connections in cervical dystonia patients and normal controls but found no significant difference. Whether decreased connectivity within the rFPN may be at least partially related to the cognitive and executive aspects or pain processing of BPS requires further exploration.

As to our knowledge, the SN has not been reported to play a role in other types of focal dystonia, such as cervical dystonia and hand dystonia, which may indicate a distinctive pathophysiology mechanism in BPS. Abnormal connections within the SN or between the SN and other regions may involve the middle temporal gyrus and the DLPFC, and these regions participate in prefrontal associational integration. The SN typically consists of the fronto-insular cortex, the dorsal ACC, the amygdala and the temporal poles. This network is believed to reflect emotional processing and to play a central role in emotional control. Recently, SN has been found to be involved in non-motor symptoms of movement disorders, e.g., mood disorders, pain, cognitive dysfunction and working memory (Metzler-Baddeley et al., 2016). Increased functional connections within the SN may be related to anxiety disorders (Pannekoek et al., 2013). Several studies have demonstrated that neuropsychiatric symptoms, particularly anxiety and obsessive-compulsive disorders, are frequent in patients with BPS (Hall et al., 2006; Fontenelle et al., 2011). Whether increased connections in the SN may be related to concomitant neuropsychiatric symptoms in patients with BPS requires further research.

In this study, we did not measure the potential dystonic activity of the orbicularis oculi musculature during scanning. However, it is known that in most patients with BPS, dystonic symptoms are absent or minimal in closed-eye states. Additionally, none of the subjects reported spasms during scanning in the post-scanning debriefings. However, this limitation must be taken into account when interpreting the results. Despite these limitations, our data provide further insights into the mechanisms underlying BPS.

In conclusion, this study demonstrated differences in multiple neural networks in primary BPS. In BPS, regions in the SMA, premotor cortex, SPL and precuneus, i.e., regions related to motor planning and execution, exhibited reduced connectivity with regard to the SMN. Selected regions in the middle frontal gyrus, DLPFC and inferior frontal gyrus areas, i.e., regions related to spatial cognition, demonstrated reduced connectivity in the right fronto-parietal network. The observation of increased connectivity of regions in the left superior frontal gyrus and middle frontal gyrus (including DLPFC) with regard to the SN is supposedly explained by the disrupted motion control.

## Author Contributions

Z-HL and H-LZ conceived and designed the experiments; X-FH, PS, C-HP and M-FN performed the experiments; X-FH, M-RZ and G-QX analyzed the data; Y-YL, Y-WM and B-WZ contributed reagents/materials/analysis tools; X-FH and M-RZ wrote the article.

## Funding

This work was supported by the Natural Science Foundation-funded Project of Liaoning Province (No. 201102052 and 2015020292).

## Conflict of Interest Statement

The authors declare that the research was conducted in the absence of any commercial or financial relationships that could be construed as a potential conflict of interest.

## Footnotes

^1^http://www.restfmri.net

^2^http://www.fil.ion.ucl.ac.uk/spm

^3^http://mialab.mrn.org/software/gift
